# Commentary: Combination surgery for ischemic cardiomyopathy: The preemptive strike

**DOI:** 10.1016/j.xjon.2021.03.014

**Published:** 2021-03-24

**Authors:** Donald D. Glower

**Affiliations:** Division of Cardiothoracic Surgery, Department of Surgery, Duke University Medical Center, Durham, NC

**Figure:**
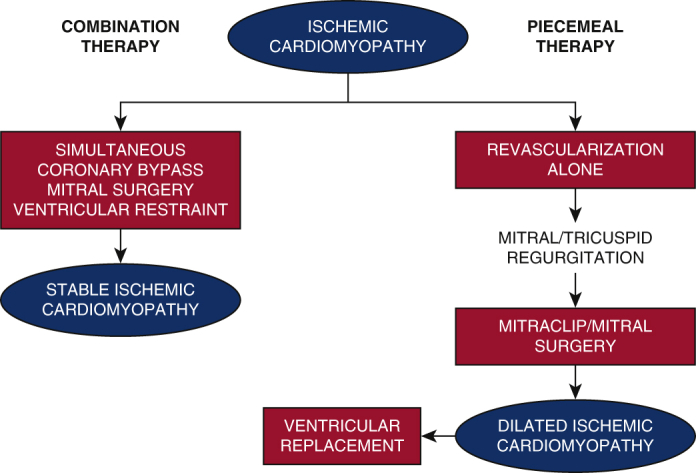
Combination therapy as an alternative to piecemeal therapy for ischemic cardiomyopathy.

**Central Message**

Combination therapy with coronary bypass, mitral surgery, and ventricular restraint therapy is proposed for ischemic cardiomyopathy.

See Article page 223.

In this issue of *JTCVS Open*, Kawabori and colleagues^1^ present a commentary to the effect that a combination of coronary artery bypass grafting, mitral valve surgery, and ventricular restraint devices may be more effective than cellular therapy for the treatment of ischemic cardiomyopathy (ICM). The authors point out that there has been limited but real success in numerous studies applying coronary artery bypass grafting, mitral valve surgery, and ventricular restraint devices individually to treat ischemic cardiomyopathy. On the other hand, human studies showing significant clinical benefit from cellular regenerative medicine to treat ischemic cardiomyopathy are lacking. The authors suggest that, like combination chemotherapy for cancer or infection, multimodality therapy for treating ischemic cardiomyopathy should be considered.

**Figure 1:**
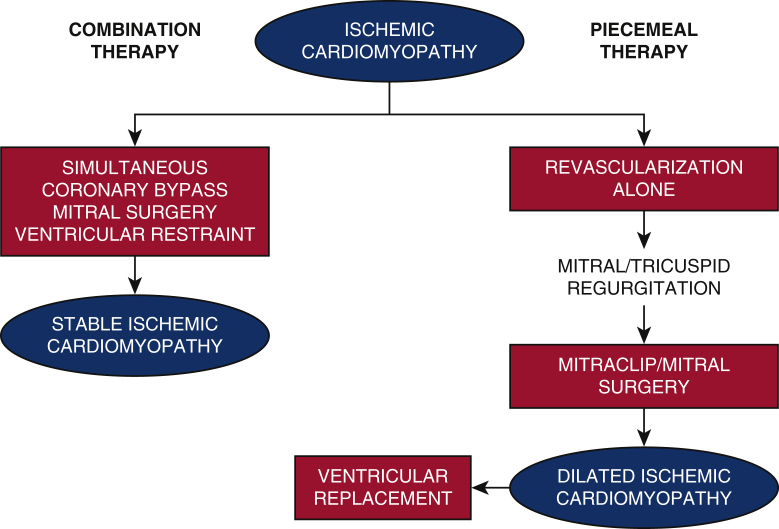
Proposed combination therapy (*left pathway*) for ischemic cardiomyopathy in which simultaneous coronary bypass, mitral surgery, and ventricular restraint therapy may produce stable ischemic cardiomyopathy. Current piecemeal therapy (*right pathway*) performs isolated revascularization, which may lead to mitral/tricuspid regurgitation, followed by mitral surgery, dilated ischemic cardiomyopathy, and possible ventricular replacement therapy.

The current piecemeal approach of multiple sequentially applied therapies for ICM theoretically has some limitations. First, application of current ventricular restraint devices can be difficult if not impossible after prior coronary bypass grafting. Second, the inherent delays between sequential therapies allows time for potentially irreversible ventricular dilation and unfavorable remodeling (Figure 1).

Surgical treatment of ischemic heart disease used to account for most of all adult cardiac surgery and 50% of all mitral surgeries in the United States.^2^ Today, treatment of ischemic heart disease is much more percutaneous than surgical with coronary stenting and transcatheter MitraClip. The push for surgical combination therapy by Kawabori and colleagues will not diminish the growing role of transcatheter treatments, but it might encourage surgeons to better define a subset of ischemic cardiomyopathy patients who can benefit from combined rather than individual surgical treatments.

The authors recognize that all ICM patients are not the same, and that selection of multimodality therapy may require “precision surgery” matching approaches to patients based on individual patient characteristics. In addition, no one stakeholder is likely to sponsor a trial combining 3 long-practiced therapies. The surgical community may need to examine combination therapy in selected patients outside of controlled trials, at least initially.

This review is more forward-looking toward future research and is not likely to change standard clinical practice immediately. However, Kawabori and colleagues do suggest a new path for investigation in combination surgical therapy for ICM. Until we have a definitive means like cellular therapy to reverse ischemic cardiomyopathy, some patients may benefit from early combined conventional revascularization, mitral intervention, and ventricular restraint therapy: the preemptive strike.
